# Identification of blood-secreted proteins as potential biomarkers for early perinatal depression

**DOI:** 10.1097/JS9.0000000000002343

**Published:** 2025-03-18

**Authors:** Sainan Duan, Xiaoqin Jiang

**Affiliations:** a Department of Anesthesiology, West China Second University Hospital, Sichuan University, Chengdu, China; b Key Laboratory of Birth Defects and Related Diseases of Women and Children (Sichuan University), Ministry of Education, Chengdu, China; c Department of Anesthesiology, West China Hospital, Sichuan University, Chengdu, Sichuan, China; d Department of Anesthesiology, Chengdu Hi-Tech Zone Hospital for Women and Children, Chengdu, China

**Highlights**

- This study emphasizes the risks associated with perinatal depression and provides strategies for its early identification rather than relying on screenings conducted after the onset of perinatal depression.
- It identifies perinatal depression in early pregnancy by analyzing four genes associated with blood-secreted proteins, while blood tests are necessary during prenatal check-ups.
- The study presents a potential mechanism for perinatal depression, which may guide future research and interventions in this field.

*Dear Editor*,

Perinatal depression, often mischaracterized solely as postpartum depression,^[^1^]^ throughout the entire perinatal period includes the whole duration of pregnancy and several weeks or up to the first year after childbirth^[^2^]^. Perinatal depression is characterized by symptoms such as fatigue, mood disturbances, and occasionally, psychotic features, occurring from pre-pregnancy through the postpartum phase. This disorder, a prevalent complication of childbirth, stands as the principal cause of perinatal mortality associated with maternal suicide^[^3^]^. The ramifications of perinatal depression extend to the infant, family, and broader societal levels.

Recent research, including a meta-analysis of randomized controlled trials published in the *International Journal of Surgery* by Wen *et al*, has demonstrated that perioperative administration of esketamine may reduce postoperative depression, with effects persisting over time for an extended period. Specifically, the esketamine group had significantly lower postoperative depression scores compared to the control group at postoperative 3 days [standardized mean differences (SMD) −1.00, 95% confidence interval (CI) −1.65 to −0.36, *P* = 0.002)], 7 days [SMD −0.59, 95% CI −0.91 to −0.26, *P* = 0.0004], and in the long term [SMD −0.42, 95% CI −0.84 to −0.01, *P* = 0.05], while esketamine significantly reduced long-term postoperative depression scores in patients with preoperative depression [SMD −0.95, 95% CI 1.81 to −0.08, *P* = 0.03]. Esketamine acted as an antidepressant by modulating the mammalian target of rapamycin complex 1 (mTORC1), which is an entity integral to immune regulation and inflammation reduction^[^4^]^. mTORC1 may emerge as a promising novel biomarker for the identification of perinatal depression. Some relevant studies have found that COX-2 inhibitors are promising for perinatal treatment^[^5^]^. While recent advances have highlighted several biomarkers potentially underpinning perinatal depression, focused research predominantly on postpartum or third-trimester phases prevails. Our study aimed to dissect the molecular pathogenesis of perinatal depression across both the gestational and postpartum periods comprehensively, seeking to delineate novel therapeutic targets.

Data were sourced from the Gene Expression Omnibus (GEO, https://www.ncbi.nlm.nih.gov/gds) database, focusing on peripheral blood gene expression profiles of 53 patients with perinatal depression and 13 controls (dataset GSE45603). Initial transcriptomic analyses during the first and third trimesters, and the postpartum period, identified differentially expressed genes (DEGs) [*P* < 0.05]. Gene Ontology (GO) enrichment analysis was conducted for on these DEGs, adopting an adjusted *P*-value threshold of less than 0.05. Subsequently, we cross-referenced these findings with data from 780 blood proteins listed in the Human Protein Atlas (HPA) database (accessed 1 September 2024, https://www.proteinatlas.org/), to discern plasma protein heterogeneity throughout the perinatal period.^[^6^]^

Fig 1A illustrates that 48 genes were differentially expressed across the first and third trimesters and the postpartum period. Fig 1B presents the 48 DEGs as most likely enriched in biological processes (BP), cellular components (CC), and the molecular functions (MF). Specifically, the primary BPs included dendritic cell differentiation and its regulation, with significant associations in the CC found the filopodium membrane and MHC protein complex, and molecular function involvement in RAGE receptor binding (Table S1. http://links.lww.com/JS9/E22). Notably, among these DEGs, HLA-G, PF4V1, S100A12, and HMGB1 are identified as blood-secreted proteins, implicated in diverse biological pathways (Figure S1, http://links.lww.com/JS9/E22). Comparative analysis revealed markedly lower expression levels of these four genes in patients with perinatal depression than in normal pregnant women at corresponding perinatal stages, with significant discrepancies observed during early pregnancy (Fig. 1C).

**Figure 1.**
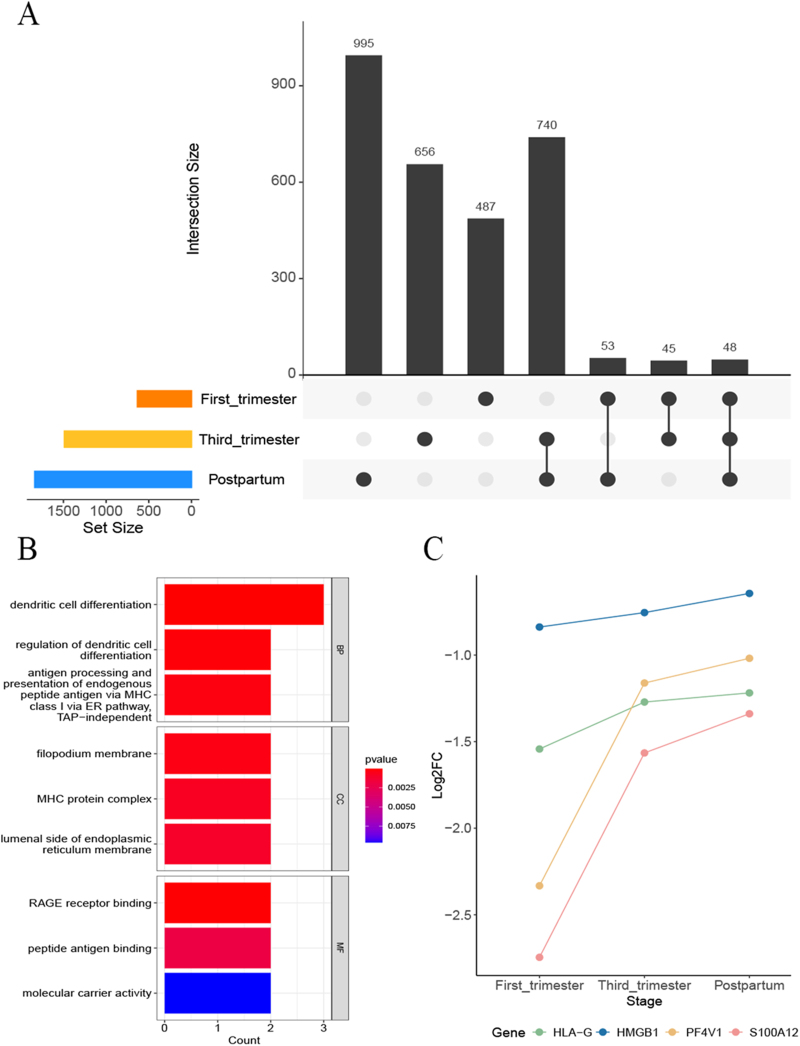
DEGs in different stages and enrichment analysis of DEGs. (A). Number of DEGs from the first trimester to the postpartum period. (B) The top three pathways identified through GO enrichment analysis (including BP, CC, and MF) of 48 DEGs. (C) The log_2_FC of 4 DEGs during pregnancy and postpartum period.

Our study successfully identified HLA-G, PF4V1, S100A12, and HMGB1 may be promising for the diagnosis of perinatal depression. Previous researches implied that perinatal depression may be associated with immune, potentially triggered by neuroinflammation and hormonal variations. Our study also confirmed that perinatal depression may be associated with immune downregulation. Perinatal depression, recognized as a significant psychological disorder, is conventionally screened post-delivery using the Edinburgh Postnatal Depression Scale (EPDS). However, the need for objective and reliable early diagnostic biomarkers remains unmet. Our findings indicated that the feasibility of preempting and mitigating perinatal depression through blood-based assays during early pregnancy, underscoring their potential integration into routine prenatal evaluations and may transform the management of maternal mental health. It is important to note that introducing blood-based biomarkers for the early identification of perinatal depression would not impose any additional trauma on pregnant women, as blood tests are already a routine part of prenatal check-ups. Generally, we propose several novel blood protein biomarkers for early detection of perinatal depression, but their robustness and generalizability will need to be further validated in larger and more diverse cohorts. Demographic, socioeconomic and other confounders need to be adjusted in the future. The impact of the COVID-19 pandemic on gene expression should also be considered^[^7^]^. Further validation through wet-bench experiments is highly warranted for our findings.

## Data Availability

Data are available from the corresponding author if justification for the requirement is Justified.
